# 213. Electronic Visit (E-visit) Use for Sexually Transmitted Infection (STI) Testing in a Large Integrated Healthcare System

**DOI:** 10.1093/ofid/ofae631.071

**Published:** 2025-01-29

**Authors:** Dana S Clutter, Amanda Thornton, Yuching Ni, Crystal Hsiao, Jonathan E Volk, Michael Silverberg, Christian Lee-Rodriguez, Mitchell N Luu, Christine B Bruno, David R Vinson, Anne Srisuro, Joshua Nugent, Jacek Skarbinski

**Affiliations:** Kaiser Permanente South San Francisco, South San Francisco, CA; The Permanente Medical Group, Vacaville, California; Kaiser Permanente Division of Research, Oakland, California; Kaiser Permanente, Pleasanton, California; Kaiser Permanente San Francisco, San Francisco, California; Kaiser Permanente Northern California, Oakland, CA; Kaiser Oakland Internal Medicine Residency Program, Oakland, CA; Kaiser Permanente, Pleasanton, California; Kaiser Permanente, Pleasanton, California; The Permanente Medical Group, Vacaville, California; The Permanente Medical Group, Vacaville, California; Kaiser Permanente, Pleasanton, California; Kaiser Oakland Department of Infectious Diseases, Oakland, CA

## Abstract

**Background:**

To improve access to sexually transmitted infection (STI) and HIV prevention services, Kaiser Permanente Northern California (KPNC), an integrated healthcare system, launched a sexual health electronic visit (E-visit) in February 2022. This E-visit allows users to self-order STI testing and other sexual health services through the healthcare patient portal.

**Figure:**
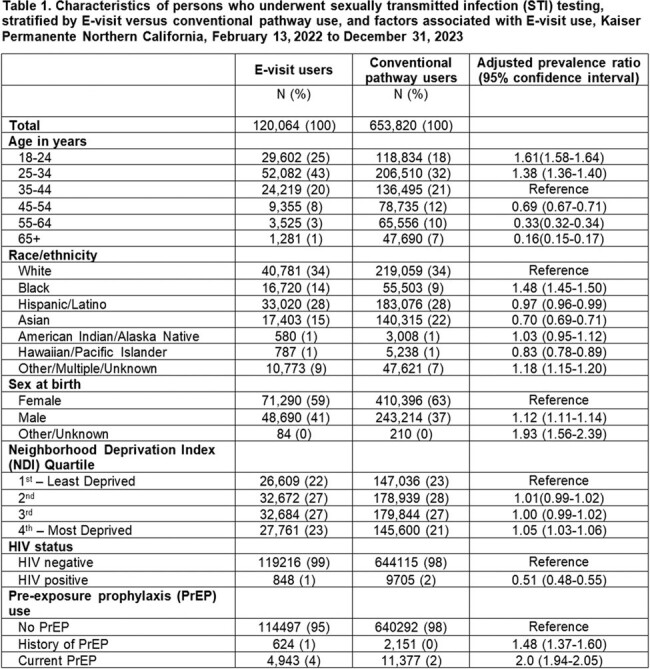

**Methods:**

We conducted a retrospective cohort study to characterize E-visit use for STI testing among all persons who had STI testing within KPNC from February 13, 2022 to December 31, 2023. We compared demographic factors of persons requesting STI testing via the E-visit with those who accessed STI services via conventional pathways. We used log-binomial regression to assess factors associated with E-visit use and compared STI test positivity among persons who used the E-visit versus those who used conventional pathways.

**Figure:**
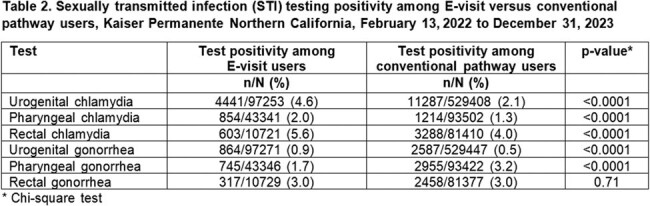

**Results:**

Of the 773,884 persons who underwent STI testing, 120,064 (16%) used the sexual health E-visit and 653,820 (84%) used conventional pathways. Younger persons were more likely to use the E-visit, with persons aged 18-24 years being the most likely to use the E-visit (adjusted prevalence ratio [aPR] 1.61 [95% CI 1.58, 1.64] compared with persons aged 35-44 years) (Table 1). Compared with white persons, black persons were more likely to use the E-visit (aPR 1.48 [95% CI 1.45, 1.5]). Men were more likely than women to use the E-visit (aPR 1.12 [95% CI 1.11, 1.14). Use was similar across socioeconomic status. Persons with HIV were less likely to use the E-visit (aPR 0.51 [95% CI 0.48-0.55]), whereas those with prior or current HIV pre-exposure prophylaxis use were more likely (aPRs 1.48 [95% CI 1.37, 1.6] and 2.0 [95% CI 1.94, 2.05], respectively). Test positivity was higher for E-visit testing compared with conventional pathways for urogenital chlamydia (4.6% vs. 2.1%; p < 0.0001), urogenital gonorrhea (0.9% vs. 0.5%; p < 0.0001), rectal chlamydia (5.6% vs 4.0% p < 0.0001), and pharyngeal chlamydia (2.0% vs 1.3%, p < 0.0001) (Table 2).

**Conclusion:**

The KPNC sexual health E-visit is used by individuals at risk for STIs, including younger and black persons, with higher test positivity for urogenital gonorrhea and chlamydia, rectal chlamydia, and pharyngeal chlamydia.

**Disclosures:**

**All Authors**: No reported disclosures

